# Supplementary material for: Possible Role of Tauroursodeoxycholic Acid (TUDCA) and Antibiotic Administration in Modulating Human Gut Microbiota in Home Enteral Nutrition Therapy for the Elderly: A Case Report
Source: Int J Mol Sci. 2024 Jun 28;25(13):7115. doi: 10.3390/ijms25137115 (PMC11240908; doi:10.3390/ijms25137115)
Supplement: Supplementary file 1 [file ijms-25-07115-s001.zip › ijms-3088511-supplementary.pdf]

Supplementary Table

Table S1. List of bacteria belonging to *Other* detected in 1C at t0 and t1

Phylum	Class	Order	Family	Genus	1C t0<br>% of mapped reads	1Ct1<br>% of mapped reads
Firmicutes	Bacilli	Bacillales	Bacillaceae	(family level ID only)	0.00	0.76
Bacteroidetes	Bacteroidia	Bacteroidales	Bacteroidaceae	(family level ID only)	0.12	0.09
Firmicutes	Clostridia	Clostridiales	Catabacteriaceae	(family level ID only)	0.00	0.07
Firmicutes	Clostridia	Clostridiales	Christensenellaceae	(family level ID only)	0.59	1.74
Firmicutes	Clostridia	Clostridiales	Clostridiaceae	(family level ID only)	2.64	5.69
Firmicutes	Clostridia	Clostridiales	Clostridiales Family XI. Incertae Sedis	(family level ID only)	0.01	0.00
Firmicutes	Clostridia	Clostridiales	Clostridiales Family XII. Incertae Sedis	(family level ID only)	0.06	0.01
Firmicutes	Clostridia	Clostridiales	Clostridiales Family XIII. Incertae Sedis	(family level ID only)	0.10	0.12
Actinobacteria	Actinobacteria	Coriobacteriales	Coriobacteriaceae	(family level ID only)	0.01	0.01
Proteobacteria	Gammaproteobacteria	Enterobacteriales	Enterobacteriaceae	(family level ID only)	20.70	0.44
Firmicutes	Erysipelotrichi	Erysipelotrichales	Erysipelotrichaceae	(family level ID only)	0.00	0.00
Firmicutes	Clostridia	Clostridiales	Eubacteriaceae	(family level ID only)	0.13	0.65
Proteobacteria	Deltaproteobacteria	Desulfuromonadales	Geobacteraceae	(family level ID only)	0.00	0.01
Firmicutes	Clostridia	Clostridiales	Gracilibacteraceae	(family level ID only)	0.07	1.00
Firmicutes	Clostridia	Halanaerobiales	Halanaerobiaceae	(family level ID only)	0.00	0.00
Firmicutes	Clostridia	Clostridiales	Lachnospiraceae	(family level ID only)	4.49	5.40
Firmicutes	Bacilli	Lactobacillales	Lactobacillaceae	(family level ID only)	0.01	0.28
Firmicutes	Clostridia	Clostridiales	Oscillospiraceae	(family level ID only)	0.51	1.80
Firmicutes	Bacilli	Bacillales	Paenibacillaceae	(family level ID only)	0.00	0.06
Firmicutes	Clostridia	Clostridiales	Peptococcaceae	(family level ID only)	0.48	2.88
Firmicutes	Clostridia	Clostridiales	Peptostreptococcaceae	(family level ID only)	0.00	0.03
Bacteroidetes	Bacteroidia	Bacteroidales	Porphyromonadaceae	(family level ID only)	2.63	0.94
Proteobacteria	Gammaproteobacteria	Pseudomonadales	Pseudomonadaceae	(family level ID only)	0.17	0.05
Bacteroidetes	Bacteroidia	Bacteroidales	Rikenellaceae	(family level ID only)	0.02	0.18
Firmicutes	Clostridia	Clostridiales	Ruminococcaceae	(family level ID only)	3.78	8.72
Synergistetes	Synergistia	Synergistales	Synergistaceae	(family level ID only)	1.06	1.10
Firmicutes	Clostridia	Thermoanaerobacterales	Thermoanaerobacteraceae	(family level ID only)	0.00	0.00
Firmicutes	Clostridia	Thermoanaerobacterales	Thermoanaerobacterales Family III. Incertae Sedis	(family level ID only)	0.00	1.23
Firmicutes	Clostridia	Clostridiales	unclassified Clostridiales	(family level ID only)	0.26	0.46
Lentisphaerae	Lentisphaeria	Victivallales	Victivallaceae	(family level ID only)	0.01	0.02
